# Combining in-situ lithography with 3D printed solid immersion lenses for single quantum dot spectroscopy

**DOI:** 10.1038/srep39916

**Published:** 2017-01-06

**Authors:** Marc Sartison, Simone Luca Portalupi, Timo Gissibl, Michael Jetter, Harald Giessen, Peter Michler

**Affiliations:** 1 Institut für Halbleiteroptik und Funktionelle Grenzflächen, Center for Integrated Quantum Science and Technology (IQ^ST^) and Research Center SCoPE, University of Stuttgart, Allmandring 3, 70569 Stuttgart, Germany; 2 4th Physics Institute and Research Center SCoPE, University of Stuttgart, Pfaffenwaldring 57, 70569 Stuttgart, Germany

## Abstract

In the current study, we report on the deterministic fabrication of solid immersion lenses (SILs) on lithographically pre-selected semiconductor quantum dots (QDs). We demonstrate the combination of state-of-the-art low-temperature *in-situ* photolithography and femtosecond 3D direct laser writing. Several QDs are pre-selected with a localization accuracy of less than 2 nm with low-temperature lithography and three-dimensional laser writing is then used to deterministically fabricate hemispherical lenses on top of the quantum emitter with a submicrometric precision. Due to the printed lenses, the QD light extraction efficiency is enhanced by a factor of 2, the pumping laser is focused more, and the signal-to-noise ratio is increased, leading to an improved localization accuracy of the QD to well below 1 nm. Furthermore, modifications of the QD properties, i.e. strain and variation of internal quantum efficiency induced by the printed lenses, are also reported.

Since the first demonstration of triggered single-photon emission^1^ in the year 2000, semiconductor quantum dots (QDs) demonstrated their strength as pure and efficient non-classical light sources. Recently, several independent demonstrations of near-unity indistinguishability^2^,^3^,^4^,^5^ have been published, showing that this technology is mature for quantum information purposes. As the QDs are embedded into a solid-state matrix, the amount of light that can be extracted is severely limited by the total internal reflection (TIR) due to the high semiconductor-to-air refractive index contrast. The first successful attempt to improve the brightness of these sources made use of planar photonic crystal structures to enhance the light extraction from a few percent up to 10–20%^6^. Several groups made efforts to further improve the light extraction by means of geometrical effects^5^,^7^,^8^,^9^, plasmonic surface interactions^10^,^11^, or by using cavity quantum electrodynamics (CQED)^3^,^4^,^12^,^13^,^14^. The use of deterministic techniques for the fabrication of such structures^15^,^16^,^17^,^18^,^19^,^20^ resulted in improved fabrication yields, thus allowing the realization of several working structures on a single chip. A macroscopic broadband approach for the light extraction from bulk semiconductors as well as from 2D materials is based on the use of solid immersion lenses (SILs) with different geometries, from hemispherical to more complex ones^21^,^22^,^23^. In the present paper we demonstrate the possibility to combine deterministic fabrication technology with micrometric-sized hemispheric SILs (h-SILs). The new approach we present for the deterministic fabrication of QD-based structures with enhanced light extraction is based on the combination of state-of-the-art low-temperature lithography^15^ with optimized femtosecond 3D laser writing^24^,^25^,^26^. This advanced fabrication procedure is demonstrated here only for QD-based sources, but can in principle be transferred to other solid-state quantum emitters.

## Methods

**Figure 1:**
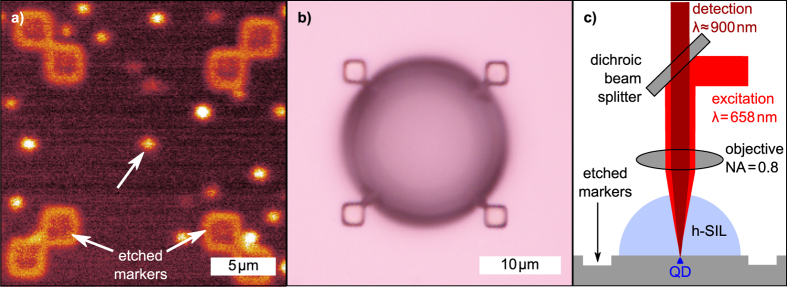
SIL fabrication and optical characterization. (**a**) μ-PL intensity map (200 × 200 pixels, acqusition time around 7 min) recorded after the etching of the deterministically positioned markers: the pre-selected QD (indicated by the white arrow) is centred between the etched alignment structures. (**b**) Microscope picture of the printed h-SIL: the markers were used as reference for the 3D printing. (**c**) Sketch of the μ-PL setup. A laser diode at 658 nm is used to excite the QD. The emitted light is collected by a dichroic beam splitter and sent into a spectrometer equipped with a CCD camera and a photon counting module.

The sample under investigation consists of a bottom distributed Bragg reflector (DBR) formed by 5 pairs of alternating AlAs and GaAs layers, followed by a GaAs spacer layer on which the InAs QDs are deposited. After an annealing step, the structure is completed with a final GaAs capping layer. The first fabrication step is based on a deterministic photolithography process at low temperature (LT), followed by 3D printing of hemispherical solid immersion lenses at room temperature (RT). Photolithography and the characterization of the sample are performed with a commercially available Attocube low-temperature lithography setup (excitation spot size ≈1 μm and collection area diameter ≈2.5 μm accordingly to specifications). Microphotoluminescence (μ-PL) maps are recorded with the aim of finding spatially isolated QDs. Furthermore, the PL spectrum is recorded in order to identify dots with narrow line widths and eventual exciton (X) and biexciton (XX) emission. Once a suitable QD is identified, high precision markers are exposed into the resist via a 532 nm laser. The exposed resist is then developed and the markers are etched in the semiconductor. The pre-selected QD results to be placed with high accuracy in the centre of the alignment markers. Figure 1(a) shows a μ-PL map after the semiconductor etching and both the selected QD and markers are simultaneously observable.

These alignment marks are also clearly visible under an optical microscope and can be used to align the 3D direct laser writer on the pre-selected QD. In comparison to conventional markers, such as crosses, the geometry of the present markers allows for a precise determination of the central location where the QD is sitting. In the second fabrication step, a commercially available Nanoscribe 3D laser writer is used. The sample is first drop coated with resist, which is exposed to the desired hemispherical geometry with submicrometric writing resolution^27^. An example of the final structure is shown in the microscope picture in Fig. 1(b). The printed h-SIL is placed symmetrically in the centre of the alignment markers. All characterizations, before and after the SIL fabrication, are performed with the same low-temperature setup. A simple sketch of the experimental setup can be seen in Fig. 1(c). The QDs are excited using a laser diode emitting at *λ*_exc_ = 658 nm, focused with a microscope objective with NA = 0.8. The emitted photons are collected using a dichroic beam splitter and sent through a single mode (SM) fibre to the spectrometer equipped with a CCD camera and a silicon-based single-photon counting module.

## Results and Discussions

**Figure 2:**
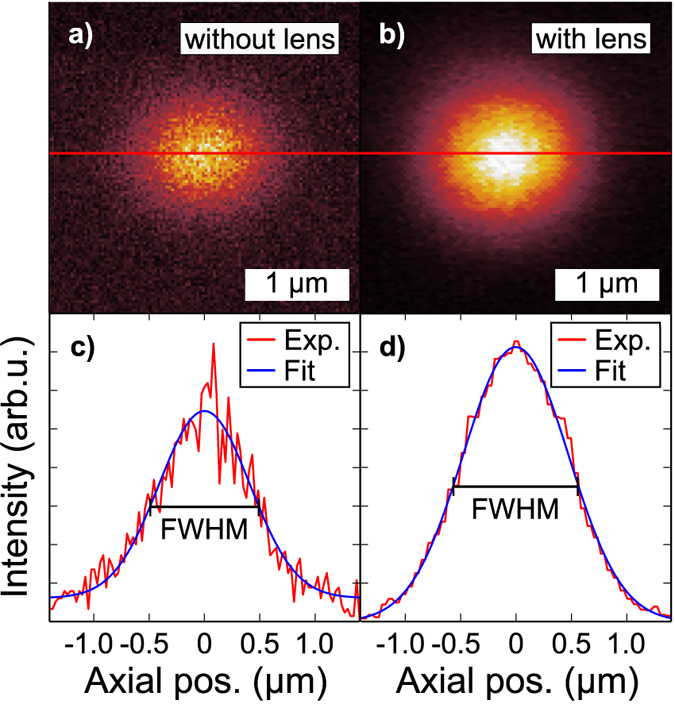
Luminescence map comparison for a single QD without and with SIL. (**a**,**b**) High resolution μ-PL maps of the same isolated single QD without and with the SIL on top. This data was used to evaluate the QD localization accuracy. (**c**,**d**) Intensity profiles (red) of maps (**a**) and (**b**), respectively, corresponding to the depicted red lines. The profile arises from the convolution of the scanning Gaussian beam over the QD. A Gaussian fit is also shown (blue).

The use of a h-SIL is known for allowing for a higher spatial magnification as well as for focusing more the laser beam^28^,^29^,^30^. In the current study, we observe both effects together with, in addition, an improved accuracy in localizing the QD. Currently, only few techniques have successfully demonstrated the deterministic placement of a photonic structure around an optically pre-selected emitter. One of the fundamental parameters that allows a reproducible procedure is the accuracy in localizing the QD before the deterministic lithography takes place. Few groups have reported success so far, using low-temperature lithography^4^,^15^, *in-situ* cathodoluminescence^16^, and recording the location of the QD with respect to prefabricated macroscopic frames, then using electron beam lithography aligned on such frames^17^. The localization accuracy of a single QD is around 20 nm^31^, 25 nm^32^, and 9 nm^17^, respectively. Thanks to an improved interferometric readout of the sample position, we are currently able to localize a QD with an accuracy better than 2 nm. This localization accuracy is further improved after printing the h-SIL. Utilizing a SIL, the incident laser beam is further focused down by a factor given by 1/*n* where *n* is refractive index of the h-SIL^28^ (in this case *n* = 1.51). On the other hand, the h-SIL also provides a magnification of the objects underneath which is proportional to the refractive index *n*. This results in a tighter focus of the laser beam and the image of the QD is magnified at the same time. As a consequence, the spatial dimension of the QD signal during acquisition of μ-PL maps remains the same as before the lens fabrication: this can be seen by comparing Fig. 2(a) and (b), where the image is given by the convolution of the scanning Gaussian beam and the QD. Analysis of the cross section of both configurations along the red line indicates, that the FWHM of the fitted 1D Gaussian curves are mostly invariant with and without h-SIL (Fig. 2(c) and (d)). Also, the modification of the TIR condition drastically improves the signal-to-noise ratio (changing the critical TIR angle from 17.1° to 26.4°), indicating a better extraction of the emitted QD light. Despite the unchanged FWHM, the improved signal-to-noise ratio results in a reduced error in localizing the QD. Choosing one single pixel line in the μ-PL data (like in Fig. 2(a) and (b)) it is not the most reliable approach for the QD localization. Indeed, the 1D Gaussian fit from such data is strongly dependent on the chosen pixel line. The most accurate approach results to be the use of a 2D Gaussian fit: the QD localization is then determined within an error of less than 2 nm without the lens and further improved by the h-SIL to 0.8 nm.

Estimating the QD location with respect to markers after the etching (instead of the aforementioned QD localization, not referred to any marker, but only to the system coordinates), like in Fig. 1(a), resulted in a conservative value of 50 to 100 nm. It is worth noting that in the present case this number is mainly limited by the accuracy in determining the edge of the etched marker in μ-PL (i.e. the convolution of the scanning beam with the marker edge) and not by the actual QD position. In any case, this value compares well with state-of-the-art techniques, but a more precise evaluation of the QD location requires a different approach in terms of marker design^32^. Even considering as an upper bound an uncertainty of 100 nm in the QD location, this incertitude is still negligible with respect to the h-SIL size.

**Figure 3:**
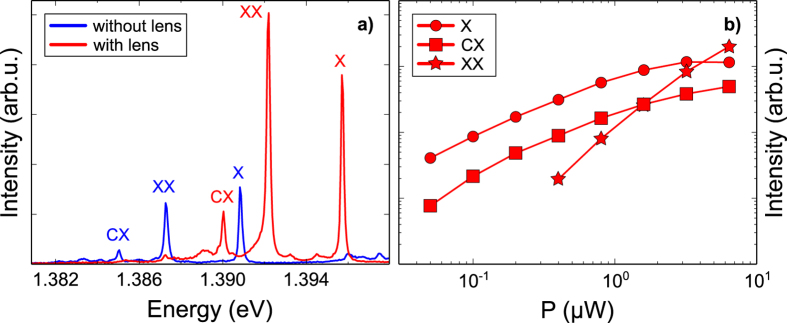
Influence on the QD’s spectral features. (**a**) Spectra of a single QD before (blue) and after (red) the fabrication of the SIL measured at X saturation. A different intensity due to enhanced light extraction is observable after the lens fabrication. Local strain effects, induced by the contraction of the SIL, are responsible for the overall spectral shift. (**b**) Power dependent intensity of the QD in (**a**) recorded after the lens fabrication.

**Figure 4:**
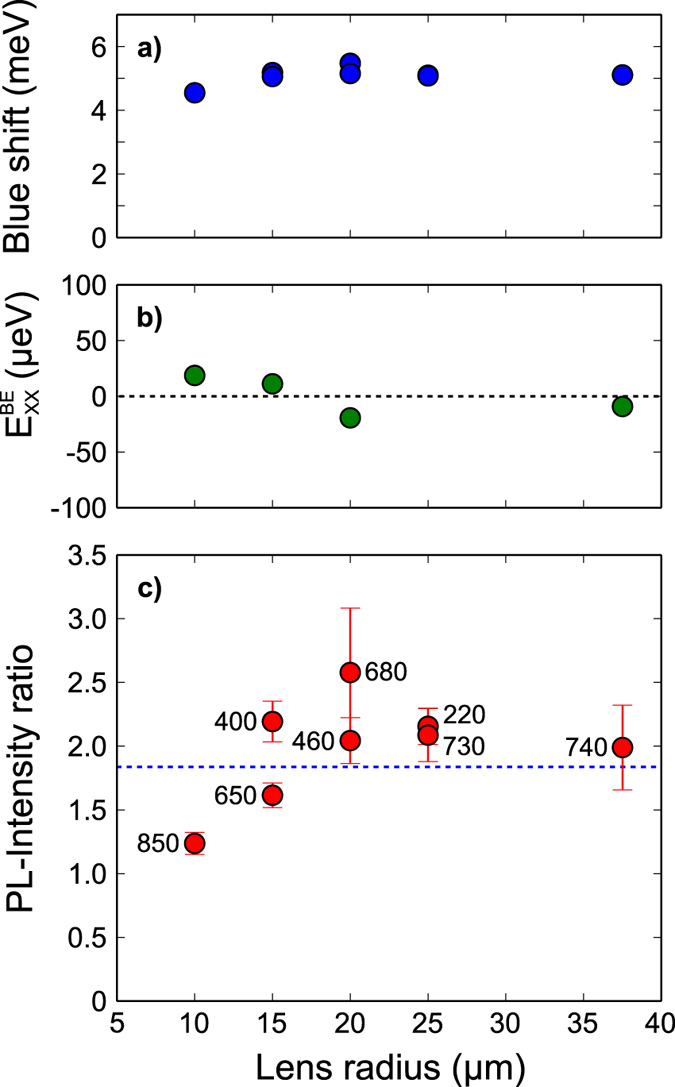
Characterization of the intensity ratio and the induced strain. (**a**) Spectral blue shift for each QD with respect to the lens radius: a comparable shift of ≈5 meV is observed. For lens radii of 15 μm, 20 μm, and 25 μm, two lenses were analysed. (**b**) Variation of the XX binding energy. The missing data points are due to a non-existent XX for some QDs. (**c**) PL-intensity ratio plotted over the lens radius. The numbers mark the refraction corrected displacement of the lens with respect to the QD (in nm). The theoretical predicted ratio of 1.84 is depicted as the blue dashed line.

As anticipated, the change in the TIR condition results into a higher light extraction. Assuming the QD as a monochromatic point source, we can expect an intensity enhancement of 1.84 (estimated taking into account the reflections at interfaces), derived from simple geometrical ray optics calculations^33^. To quantify such an enhancement, a direct spectral comparison before and after the h-SIL fabrication has been performed. Figure 3(a) depicts two μ-PL spectra of the same QD before and after the lens fabrication. A clear enhancement of all spectral lines, indicating the SIL to be nearly achromatic in the current wavelength range, together with an overall blue shift of all spectral features is observed. The quasi-particle states are identified via power-dependent measurements, as displayed in Fig. 3(b). The observed spectral shift is a common feature when a QD undergoes local strain. In the present case, the h-SILs are printed at room temperature and then cooled down to 4 K during the measurements. This results in a compressive strain applied to the QD. The spectral shift for all dots under study is comparably around 5 meV (Fig. 4(a)), allowing us to estimate an applied stress of around 180 Mpa^34^,^35^. When InAs QDs undergo biaxial strain, a variation of the XX binding energy is generally observed. This is what we expect also in the present case, considering the hemispherical shape of the SIL. Figure 4(b) reports values of variation of such binding energy around ±20 μeV, lower than previously observed for a comparable applied biaxial strain^34^. This difference can be explained by a change of the dot stoichiometry, that could result in a different dot behaviour under applied strain.

As anticipated, the h-SIL leads to an increase in the angle of total internal reflection, resulting in an increase of the extracted light from the GaAs layer. However, the estimation of the enhancement requires further clarification due to the fact that the relative intensity between X and CX states often varies before and after SIL fabrication. A different power spectral density can affect trapped charges, consequently modifying the internal quantum efficiency^36^. We ascribe such a change to the tighter focusing of the excitation laser beam. This implies, that the PL-intensity ratio has to be defined as


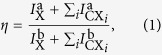


with *I*_X_ as the exciton intensity and 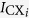 the intensity of the i-th charged state. The superscripts (a) and (b) indicate the intensities after or before the SIL fabrication respectively. Figure 4(c) reports the measured PL-intensity ratios for all dots under investigation, with relative error bars. The overall behaviour (in average *η* = 1.99 ± 0.21) agrees well with the calculated expected values of 1.84. The relative displacement (in nm) of each lens with respect to the corresponding QD is also reported in the figure, as the number beneath each experimental point. All lenses are positioned with submicrometric accuracy. The only deviation from the calculated extraction efficiency, appears to happen for small lens radii, even being the displacement comparable to other structures. This could be related to the fact that for the same lens displacement, the incident and extracted beams would perceive a different lens curvature, which is larger for small SIL radii. The SIL placement is then critical only for small radii. Despite that, it is worth noticing that radii exceeding 40 μm are not suitable for LT experiments. At the current lens geometry, the SILs do not survive the cooling cycle and crack.

**Figure 5:**
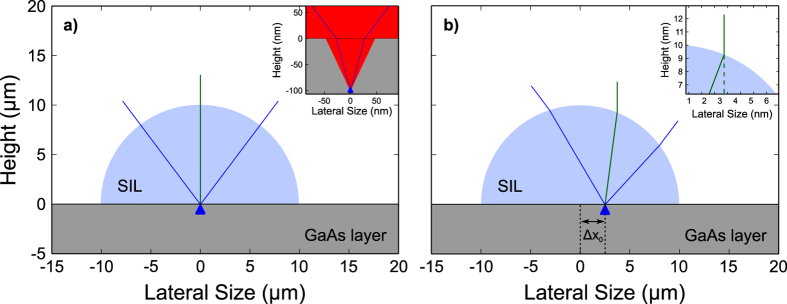
Geometrical description of the light collected by the current configuration. (**a**) depicts a hemispheric lens placed exactly on top of the QD and the blue lines represent the experimental NA = 0.8. The green line depicts the light beam exiting the lens orthogonally to the GaAs layer. The inset shows the emitted light that can be extracted from the semiconductor (calculations performed with h-SIL). For larger emission angles, TIR conditions are fulfilled so the light is confined into the GaAs layer. Only a fraction of this light can be collected with the present NA (blue lines). (**b**) QD displaced with respect to the centre of the h-SIL. For clarity a displacement value of 2.5 μm (more than 3 times larger that the maximum observed displacement) is chosen. Ray optics have been used for calculating the beam refraction at the lens interface. The green line shows the beam leaving the lens orthogonally to the GaAs surface. For sake of completeness, the maximum collected angle is also depicted (blue).

The numbers in Fig. 4(c) are extracted from the measurement of the displacement, corrected accounting for the light extraction. The centre of the SIL has been determined by imaging laser reflection onto a CCD camera: when impinging on the centre of the SIL, the reflected light displays a symmetric diffraction pattern. The laser is then displaced to maximize the PL signal and the coordinates are recorded with respect to the SIL centre. The measured values are reported in the second column of Table 1. On the other hand, these numbers do not represent the actual lens displacement, since one has to consider how the image is modified by the SIL itself. Figure 5(a) depicts the case where the lens is placed exactly on top the the QD. Using ray optics it is then possible to calculate the amount of light collected by the current NA (0.8), giving the aforementioned factor of 1.84. The central green line represents the ray leaving the lens orthogonally to the GaAs layer. On the other hand, if the lens is displaced from the QD position as depicted in Fig. 5(b), the maximum PL signal is measured for a position that does not correspond to the actual QD position. Indeed the ray that exits the lens orthogonally to the GaAs layer, i.e. the maximum of the Gaussian-shaped PL signal in Fig. 2(b), has been refracted by the SIL, then coming from a different sample location, closer to the centre of the lens (see inset Fig. 5(b)). Once more using geometrical ray optics, the measured displacement values can be corrected, compensating for the beam refraction, giving rise to the values in the third column of Table 1: utilizing the current 3D printing fabrication technique, submicrometric accuracy in placing the lens can be obtained. At the moment, the limiting accuracy factor in the lens placement is the alignment of the 3D laser writing machine on the etched markers, which could be improved in the future by depositing chromium or gold as markers instead of etching.

## Conclusion

In the present work we demonstrated a novel procedure to combine low-temperature lithography with 3D direct laser writing. The photolithography technique is utilized for localizing a single QD with an accuracy better than 2 and then deterministically fabricating alignment markers around it. These markers are then used to align a 3D laser printer, which allows for the fabrication of deterministically placed hemispheric SILs with submicrometric accuracy. In addition to the enhanced extraction of the emitted QD light due to the modified TIR condition, the excitation laser spot is scaled down with a factor of 1/*n* while the PL image of the QD is magnified by the lens by a factor of *n*. The physical effects induced by such lenses are also discussed: the thermally-induced contraction of the polymer while cooling to 4 K results in a local strain on the QD, which leads to an overall spectral shift of the emitted photons. The spot size reduction further modifies the carrier dynamics. This results in a variation of the internal quantum efficiency that reflects in a different ratio between neutral and charged states with and without the SIL. Considering that, the total light extracted using the h-SIL is enhanced by a factor of ≈2 with respect to the bare sample. This value agrees well with the calculated enhancement factor. The use of the current geometry allows for an enhanced extraction efficiency mostly independent from the emitter wavelength. This could be interesting for applications, which require the exciton and biexciton to be simultaneously enhanced. On the one hand, a large hemispherical SIL is not particularly sensitive to the placement with respect to the emitter position. This is the reason why several groups simply glued macroscopic lenses on the sample surface. On the other hand, the use of micro-sized objects is of interest for micro-optics purposes, where small-sized samples are used (strain tuned or electrically contacted samples.) Reducing the size of the SIL requires a higher precision in placement that becomes more and more critical for structures like super SILs, where the reciprocal lens-to-emitter positioning is fundamental to produce an efficiently working device.

The present results open the route to the use of more complex structures for light extraction as well as for applications in which the emitter position with respect to the printed device is crucial. The present lithography procedure, i.e. writing deterministically-placed alignment markers, can be in principle used in combination with other lithography techniques such as e-beam lithography, that would allow for nanometric writing resolution around the pre-selected QD. Deterministically placing a SIL on top of an emitter could also be used to further enhance the resolution of laser lithography^28^ for writing more precise structures around a pre-selected location with an emitter localization accuracy below 1nm.

## Additional Information

**How to cite this article**: Sartison, M. *et al*. Combining in-situ lithography with 3D printed solid immersion lenses for single quantum dot spectroscopy. *Sci. Rep.* **7**, 39916; doi: 10.1038/srep39916 (2017).

**Publisher's note:** Springer Nature remains neutral with regard to jurisdictional claims in published maps and institutional affiliations.

## Figures and Tables

**Table 1:** Displacement of the printed SILs with respect to the QD position.

radius (μm)	measured (nm)	refraction corrected (nm)
10	1350	850
15	510	400
15	900	650
20	860	460
20	1210	680
25	330	220
25	1350	730
37.5	1270	740

Refraction corrected values show submicrometric accuracy.
